# 59473 Clinical and demographic predictors of the need for pharmacotherapy in Neonatal Abstinence Syndrome (NAS)

**DOI:** 10.1017/cts.2021.771

**Published:** 2021-03-30

**Authors:** Shawana Bibi, Janis Breeze, Norma Terrin, Jonathan Davis

**Affiliations:** 1 Tufts Children’s Hospital; 2 Tufts Clinical and Translational Science Institute

## Abstract

ABSTRACT IMPACT: This work has the potential to help clinicians decide which infants exposed to in utero opioids, will need to be treated early or can be discharged home early based on their risk, thus reducing prolonged hospitalization OBJECTIVES/GOALS: To develop and validate a prediction model with inclusion of clinical and demographic risk factors to identify infants with NAS likely to need pharmacotherapy. METHODS/STUDY POPULATION: A pooled cohort of 761 infants from 5 different studies including 2 trials and 3 observational cohorts will be used to develop the model.

All infants >than or equal to 37 weeks gestational age born to mothers with history of OUD will be included. Infants with congenital disorders and severe medical and surgical illnesses will be excluded. Multivariable mixed effects logistic regression modeling will be performed to predict the need for pharmacologic treatment for NAS. Candidate variables will be included based on clinical knowledge and previously published data. Model performance will be evaluated by measuring discrimination using Area Under the Curve (AUC) statistics and calibration. Model will be internally validated using boot strap validation. RESULTS/ANTICIPATED RESULTS: Pending data analysis DISCUSSION/SIGNIFICANCE OF FINDINGS: Opioid Use Disorder in pregnancy has resulted in concurrent rise in NAS incidence. NAS affects opioid exposed infants variably and accurate prediction of its severity and need for treatment remains elusive. Known clinical and demographic factors can predict the need for NAS therapy in opioid exposed infants, aiding clinical decision making.

